# A nomogram prediction model for 30-day mortality risk in severe cerebral infarction patients integrating inflammation-related indicators and APACHE II score

**DOI:** 10.3389/fneur.2026.1918006

**Published:** 2026-09-23

**Authors:** Zhou Shu, Fengbo Fu, Jinyi Tian

**Affiliations:** 1 Department of Intensive Care Medicine, Chongqing University Qianjiang Hospital, Chongqing, China; 2 Department of Intensive Care Medicine, the First Affiliated Hospital of Changsha Medical University, Changsha, Hunan, China; 3 Department of Neurology, Chongqing University Qianjiang Hospital, Chongqing, China

**Keywords:** acute physiology and chronic health evaluation score, inflammatory indicators, logistic regression, mortality risk, severe cerebral infarction

## Abstract

**Objective:**

To explore independent risk factors for 30-day all-cause mortality in severe cerebral infarction and construct a nomogram prediction model incorporating inflammation-related indicators and APACHE II score.

**Methods:**

A total of 312 patients with severe cerebral infarction from Chongqing University Qianjiang Hospital and 134 external validation cases from the First Affiliated Hospital of Changsha Medical University were retrospectively analyzed. Demographic, comorbidity, laboratory, and APACHE II data were collected from all patients. The endpoint was 30-day all-cause mortality. Univariate and multivariate logistic regression were used to identify independent risk factors, and a nomogram was developed.

**Results:**

In this cohort, age distribution differed notably between the two subgroups: participants who did not survive had a higher mean age than those who survived. Regarding clinical and laboratory markers, non-survivors exhibited elevated NIHSS scores, NLR, hs-CRP, IL-6 levels, and APACHE II scores when compared with survivors, and the between-group differences were statistically significant (*p* < 0.05). After adjusting for confounding factors, multivariate logistic regression modeling further identified that NIHSS score, NLR, hs-CRP, IL-6, and APACHE II score were independent factors linked to fatal outcomes in cases of severe cerebral infarction (all *p* < 0.05). The ROC curve analysis showed that the combined use of the NIHSS, hs-CRP, IL-6, NLR, and APACHE II scores had a higher AUC value for predicting mortality in patients with severe cerebral infarction than individual tests (*Z* = 6.879, 6.454, 6.693, 5.480, 5.407, *p* < 0.05). Based on Logistic regression analysis, a prediction nomogram for the risk of 30-day mortality in patients with severe cerebral infarction was established. The nomogram included five variables, NIHSS score, NLR, hs-CRP, IL-6, and APACHE II score, and this model had high calibration ability and clinical practicability.

**Conclusion:**

NIHSS, NLR, hs-CRP, IL-6, and APACHE II are independent risk factors for 30-day mortality in patients with severe cerebral infarction. The integrated model offers excellent predictive performance, aiding early risk screening and personalizing treatment.

## 1 Introduction

Severe cerebral infarction is the leading neurological emergency that causes death and long-term disability in adults worldwide. It requires comprehensive treatment in the neuro-intensive care unit (NICU). Even under standard treatment protocols, the early mortality rate of patients with severe cerebral infarction is 20% to 40% (1). Early precise assessment of the risk of death is of great significance for optimizing the allocation of medical resources, strengthening the monitoring of high-risk populations, and promptly adjusting treatment strategies. The Acute Physiology and Chronic Health Evaluation II (APACHE II) is one of the most widely used disease severity assessment systems in the intensive care unit. It comprehensively reflects the degree of acute physiological disorder and the basic health status of patients. Its value in the prognosis assessment for various critically ill patients has been confirmed in numerous studies (2). However, cerebral infarction has a unique pathophysiological mechanism. Simply relying on APACHE II is insufficient to fully capture the systemic inflammatory response and immune disorder characteristics after neurological damage. Recent studies have shown that cerebral tissue ischemia–reperfusion injury triggers intense sterile inflammatory responses. Dynamic changes in inflammatory mediators and immune cells in the peripheral blood not only reflect the severity of brain injury but also directly participate in the occurrence of secondary brain injury and multiple organ dysfunction (3). Among them, indicators such as the neutrophil-to-lymphocyte ratio (NLR) and high-sensitivity C-reactive protein (hs-CRP), which are convenient to measure and low in cost, have been gradually incorporated into the prognostic stratification of stroke patients (4, 5). However, studies that integrate these indicators representing neuroinflammation with general critical illness scoring systems to predict the mortality risk of specific populations with severe cerebral infarction more accurately are still limited. The nomogram can graphically and quantitatively present the results of logistic regression, enabling individualized calculation of risk probabilities. In recent years, it has been widely used for the construction of clinical prediction models. This study aimed to integrate the inflammatory-related indicators at the beginning of hospitalization and the APACHE II score to construct a simple, intuitive, and highly accurate nomogram model for predicting the 30-day in-hospital mortality risk of patients with severe cerebral infarction, with the expectation of providing high-quality evidence for clinical decision-making.

## 2 Data and methods

### 2.1 Research objects

We retrospectively reviewed the clinical records of 312 inpatients diagnosed with severe cerebral infarction who received treatment at Chongqing University Qianjiang Hospital between January 2023 and December 2025. Meanwhile, 134 eligible patients treated at the First Affiliated Hospital of Changsha Medical University over the same timeframe were enrolled to form the external validation cohort. The inclusion and exclusion criteria for the validation group were identical to those for the model group. The inclusion criteria were as follows: (1) met the diagnostic criteria stipulated in the “Chinese Guidelines for the Diagnosis and Treatment of Acute Ischemic Stroke” (6) and confirmed by head CT or MRI; (2) admitted to the intensive care unit within 24 h of onset; (3) had a National Institutes of Health Stroke Scale (NIHSS) (7) score of ≥15 at admission; and (4) had complete clinical data. Consequently, there were no missing values for any of the variables included in the final analyses. The requirement for complete data was implemented to ensure data integrity and avoid potential biases associated with imputation methods in our relatively small sample. The exclusion criteria were as follows: (1) hemorrhagic stroke or mixed stroke; (2) pre-existing severe liver or kidney dysfunction, malignancy, hematological diseases, or autoimmune disorders; (3) recent history of infection or treatment with immunosuppressants; and (4) pregnancy or lactation. Participant flow diagram see Figure 1.

**Figure 1:**
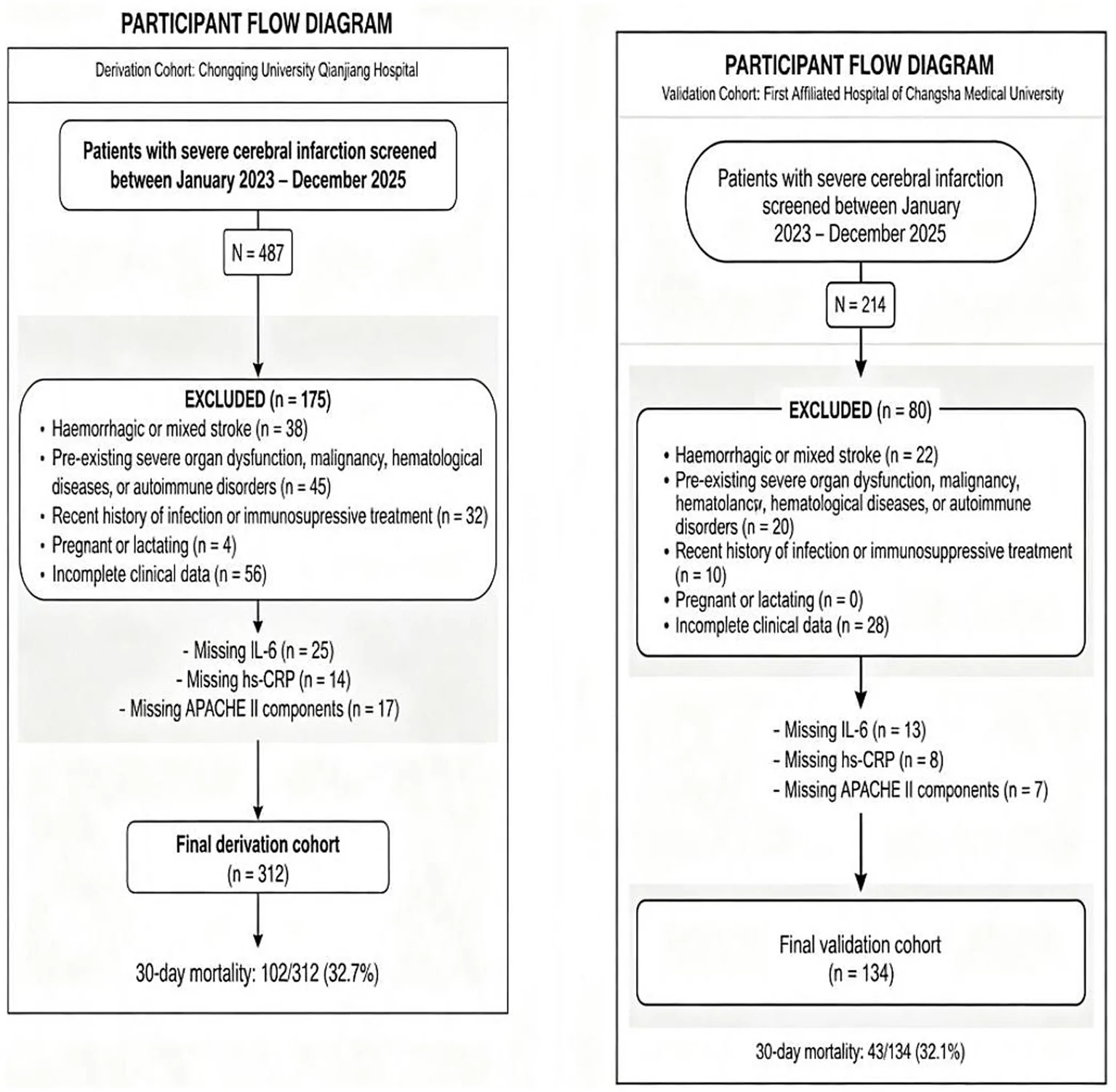
Participant flow diagram.

Rationale for exclusion criteria: Patients with pre-existing severe organ dysfunction, malignancies, haematological diseases, or autoimmune disorders were excluded to minimize potential confounding factors from chronic systemic inflammation, which could independently influence NLR, hs-CRP, and IL-6 levels. Patients with a recent history of infection or immunosuppressive treatment were excluded to ensure that the measured inflammatory markers primarily reflect stroke-related neuroinflammation rather than pre-existing or concurrent infectious or immunomodulatory processes. Although we acknowledge that these exclusion criteria may limit the generalizability of our model to unselected neurocritical care populations, they were considered necessary to establish the biological validity of the prognostic model in a well-defined study population. The pre-experiment of this study showed that the 30-day mortality rate in patients with severe cerebral infarction was approximately 30%. Six potential independent variables were included. Based on the calculation of EPV = 15, the minimum number of outcome events required was 6 × 15 = 90, corresponding to the required total sample size of approximately 90/0.3 = 300. The final inclusion of this study in the model group was 312 cases, among which 102 died, with EPV = 102/6 = 17, meeting the requirement of EPV ≥ 10. The validation group included 134 cases, among which 43 died, also meeting the sample size requirement of the validation set.

### 2.2 Data collection instruments

#### 2.2.1 Participant information sheet

Relevant clinical records were obtained from the enrolled patients. The main parameters included age, sex, body mass index (BMI), and prior medical history such as hypertension, diabetes, coronary artery disease, and atrial fibrillation. Smoking history was defined as a continuous or total smoking duration of ≥ 6 months, while drinking history referred to regular alcohol intake: no less than three times per week, ≥20 g ethanol per time, for at least 1 year. We further documented the admission NIHSS and Glasgow Coma Scale (GCS) scores (8). We also noted whether patients received mechanical ventilation or endovascular reperfusion treatments, namely intravenous thrombolysis, intra-arterial thrombolysis, mechanical thrombectomy, and stent placement. Infarct sites and associated complications were also noted.

#### 2.2.2 Laboratory observation index testing

All blood samples were collected within 2 h of ICU admission, prior to the initiation of any major therapeutic interventions (including mechanical ventilation, thrombolysis, or endovascular therapy). The samples were immediately sent for testing and were completed within 30 min. The samples were analyzed using the Sysmex XN-2000 automatic blood analyzer and its corresponding reagents to determine the white blood cell count (WBC), neutrophil count (NEU), lymphocyte count (LYM), monocyte count (MON), and platelet count (PLT). Three milliliters of gel-stabilized venous blood was separated and centrifuged at 3000 rpm for 10 min to obtain serum. A Beckman Coulter AU5800 automatic biochemical analyzer and its corresponding reagents were used to detect high-sensitivity C-reactive protein (hs-CRP), procalcitonin (PCT), alanine aminotransferase (ALT), aspartate aminotransferase (AST), total bilirubin (TBIL), serum creatinine (Cr), blood urea nitrogen (BUN), and fasting blood glucose (FBG) levels. A Roche Cobas e601 automatic electrochemiluminescence immunoassay analyzer and corresponding reagents were used to detect interleukin-6 (IL-6) levels. All tests were performed in accordance with the instrument and reagent guidelines. The results of the internal quality control tests were within the allowable range. Based on the above blood routine test results, a dedicated person calculated the NLR using the formula NLR = NEU/LYM. All original test data and calculated results of the derived inflammatory indicators were retained to one decimal place and double-checked by two people to ensure data accuracy.

The intended prediction time for this model was the time of ICU admission. Accordingly, all predictor variables (NIHSS, NLR, hs-CRP, IL-6, and APACHE II scores) were measured or calculated based on data available within the first 4 h of ICU admission and prior to the initiation of major therapeutic interventions. Variables representing subsequent treatments (e.g., mechanical ventilation, endovascular therapy) or in-hospital complications (e.g., stroke-associated pneumonia, symptomatic cerebral edema, gastrointestinal bleeding) were recorded for descriptive purposes only. These variables were strictly excluded from the candidate predictor pool in both univariate and multivariate regression analyses. This exclusion was performed to eliminate any possibility of temporal bias and outcome leakage, as these events occur after the baseline prediction time point (ICU admission) and may be on the causal pathway between the baseline risk and the outcome.

#### 2.2.3 APACHE II score

As described in reference (9), the APACHE II scoring system falls into three major sections: acute physiological assessment, age-related scoring, and chronic health evaluation. The total possible points range from 0 to 71. In general, higher total points reflect a worse clinical status and less favorable long-term outcomes. The acute physiological part covers 12 physical parameters, including body temperature, mean arterial pressure, and heart rate. Each item is graded from 0 to 4 points, with scores determined by the extent of derivation of measured value from the normal reference range. If acute renal failure occurs due to acute kidney injury, the score is doubled. To accurately reflect the initial degree of acute physiological disorder at admission and avoid the interference of early treatment interventions, the APACHE II score was calculated based on the worst physiological parameters recorded during the first 4 h of ICU admission, before the implementation of major interventions such as mechanical ventilation, sedation, dehydration, or vasoactive drugs. This approach ensured that the score reflected the patient’s baseline physiological status at the intended prediction time point. If an indicator was not tested before treatment, the worst value within 24 h of admission without corresponding treatment intervention was recorded. The age score was scored based on the patient’s age in segments: ≤ 44 years old: 0 points; 45–54 years old: 2 points; 55–64 years old: 3 points; 65–74 years old: 5 points; ≥ 75 years old: 6 points. The chronic health score added 5 points if the patient had a history of severe organ dysfunction or immunodeficiency and was a non-surgical patient or a patient after elective surgery. If the patient underwent emergency surgery 2 points were added. Severe organ dysfunction included NYHA class IV cardiac function, severe chronic lung disease, long-term dialysis treatment, liver cirrhosis with portal hypertension or upper gastrointestinal bleeding history, and receiving immunosuppressant chemotherapy radiotherapy or having an immunodeficiency disease. The APACHE II score is the sum of the scores of the three parts. Two intensivists who had received specialized training in this scoring system independently scored the patients. In the case of inconsistent scores, a senior physician with more experience reviewed and reached a consensus. The final consistent result was obtained.

### 2.3 Outcome indicators

The primary endpoint was defined as 30-day all-cause mortality from the date of ICU admission. The date of death for patients who died during hospitalization was obtained from electronic medical records. For patients who were discharged or transferred before the 30-day mark, their survival status was ascertained through a structured telephone follow-up conducted on day 30 (± 2 days) after ICU admission by trained research nurses blinded to the baseline predictor values. Patients who could not be contacted were considered lost to follow-up; however, the 30-day follow-up was complete for all 312 (100%) patients in the derivation cohort and for all 134 (100%) patients in the validation cohort. No patients were lost to follow-up in this study. Based on the primary endpoint, 312 patients were divided into the survival (*n* = 210) and death (*n* = 102) groups.

### 2.4 Statistical methods

All statistical calculations were performed using SPSS 26.0 and R 4.2.1 software. Enumeration data are presented as cases and percentages [*n* (%)], and the chi-square test was used for intergroup comparisons. The Kolmogorov–Smirnov (K-S) test was applied to examine whether the datasets conformed to normal distribution. Variables with normal distribution are summarized as mean ± standard deviation (*x̄* ± s). For between-group comparisons, an independent t test was used for normally distributed indicators. For data that did not follow a normal distribution, results are reported using median and interquartile range, namely M (P₂₅, P₇₅), and the Mann–Whitney U test was selected for statistical comparison. The multicollinearity was tested using the variance inflation factor (VIF), and it was considered to have no significant multicollinearity if VIF was less than 5.

Multivariable logistic regression analysis was performed to identify the risk factors for mortality in patients with severe cerebral infarction. To control for potential confounding factors and identify independent risk factors, the forward stepwise likelihood ratio (LR) method was used for variable selection. All baseline variables (i.e., those collected at or before the prediction time point) with a *p*-value < 0.10 in the univariate analysis were entered into the multivariable regression model. Post-admission variables, including therapeutic interventions and complications, were not included as candidate predictors in this step to prevent temporal bias. However, given its clinical importance as a potential confounder, endovascular therapy was forced into the multivariable model as a covariate, but this should be interpreted as a confounder adjustment rather than a predictive variable for the nomogram. We acknowledge that stepwise regression has potential limitations, including model instability and the risk of overfitting. However, this approach was considered appropriate for the present study for several reasons: (1) the number of candidate variables was limited to six, and with 102 outcome events, the events-per-variable ratio was 17, well above the recommended minimum of 10, thereby substantially reducing the risk of overfitting; (2) this study was primarily exploratory, aiming to identify clinically interpretable independent risk factors rather than to develop a high-dimensional model for maximum predictive accuracy; and (3) the forward LR method was used, which is more conservative than backward elimination.

Given its clinical importance as a potential confounder, endovascular therapy was forced into the multivariable model as a covariate to adjust for its potential effect on the outcome, regardless of its statistical significance in the univariate analysis. The assumption of linearity in the logit for all continuous predictors was assessed using restricted cubic splines (RCS) with three knots. Tests for nonlinearity were not statistically significant for any of the final predictors (all *p* > 0.05), supporting their inclusion as linear terms in the final model. Influential observations and outliers were assessed using Cook’s distance and studentized residuals; no influential observations exceeding the predefined threshold (Cook’s distance > 1) were identified. Clinically plausible interactions among key predictors were also explored, but none were statistically significant.

The receiver operating characteristic (ROC) curve was used to assess how well these markers could predict mortality in patients with severe cerebral infarction. The difference in AUC was compared using the DeLong method. Internal validation was performed using 1,000 bootstrap resampling. Within each bootstrap sample, the entire model development procedure, including univariate screening and stepwise variable selection, was repeated. The model performance was evaluated to estimate and correct for optimism. The optimism-corrected AUC, calibration slope (ideal = 1), calibration intercept (ideal = 0), and Brier score were reported as overall measures of prediction error. A well-calibrated model has a calibration slope close to 1 and an intercept of approximately 0. The Brier score ranged from 0 to 1, with lower values indicating better predictive accuracy.

DCA was used to assess the practical clinical value of the prediction model. The relevant clinical net benefits at varying threshold probability levels were calculated. Moreover, DCA curves were generated to contrast the net benefits derived from the model prediction, comprehensive intervention, and non-intervention strategies. For external validation, the fixed regression coefficients and intercepts from the final model developed in the derivation cohort were applied directly to the validation cohort to calculate the predicted probability of mortality for each patient. No re-estimation or recalibration of the original model coefficients was conducted. Model performance in the validation cohort was assessed using the AUC with its 95% confidence interval, calibration intercept, calibration slope, observed-to-expected (O: E) event ratio, and Brier score. A calibration plot was generated to visually assess the agreement between the predicted and observed outcomes. To rigorously validate the robustness of our findings against the potential limitations of stepwise selection, a pre-specified full model was constructed by forcing all clinically relevant candidate predictors (age, NIHSS, NLR, hs-CRP, IL-6, APACHE II, and GCS) into the logistic regression model without applying any variable selection. To mitigate overfitting and evaluate model stability, we performed internal validation using 1,000 bootstrap resamples. Model performance (AUC and calibration) of this full model was then compared to our stepwise model. The significance level α was set at 0.05.

## 3 Results

### 3.1 Comparison of general data between the two groups

Patients in the death group was older and had higher NIHSS scores, NLR, hs-CRP, IL-6 levels, and APACHE II scores than those in the survival group (*p* < 0.05; Table 1).

**Table 1:** Comparison of general data between the two groups.

Index	Survival group (*n* = 210)	Death group (*n* = 102)	*t/χ*^2^/*Z* value	*p* value
Age (years)	65.43 ± 11.03	68.20 ± 11.04	2.083	0.038
Male	125 (59.24)	57 (56.44)	0.375	0.541
BMI (kg/m^2^)	24.10 ± 2.97	24.11 ± 2.86	0.035	0.972
History of hypertension	146 (69.19)	74 (72.55)	0.302	0.583
History of diabetes mellitus	72 (34.12)	43 (42.16)	1.828	0.176
History of coronary heart disease	49 (23.22)	30 (29.41)	1.341	0.247
History of atrial fibrillation	42 (19.91)	26 (25.74)	1.214	0.271
Smoking history	87 (41.23)	49 (48.04)	1.220	0.269
Drinking history	65 (30.81)	40 (39.22)	2.100	0.147
NIHSS score (points)	20.26 ± 3.94	22.33 ± 3.76	4.416	<0.001
GCS score (points)	8.50 ± 2.23	8.26 ± 2.23	0.905	0.366
Mechanical ventilation	98 (46.45)	57 (55.88)	2.332	0.127
Endovascular therapy	62 (29.38)	27 (26.73)	0.314	0.575
Infarct location			0.888	0.346
Anterior circulation	147 (70.00)	66 (64.71)		
Posterior circulation	63 (30.00)	36 (35.29)		
Complications				
Stroke-associated pneumonia	82 (39.05)	47 (46.08)	1.399	0.237
Symptomatic cerebral edema	74 (35.24)	41 (40.20)	0.725	0.394
Gastrointestinal bleeding	32 (15.24)	19 (18.63)	0.577	0.448
WBC (×10^9^/L)	11.46 ± 2.90	11.06 ± 2.56	1.184	0.237
MON (×10^9^/L)	0.67 ± 0.18	0.70 ± 0.19	1.085	0.279
PLT (×10^9^/L)	220.76 ± 55.05	213.67 ± 51.96	1.086	0.278
NLR	7.79 ± 2.38	10.06 ± 2.51	7.756	<0.001
hs-CRP (mg/L)	26.87 ± 8.04	32.23 ± 7.73	5.591	<0.001
IL-6 (pg/mL)	45.16 ± 12.70	54.00 ± 14.72	5.473	<0.001
PCT (ng/mL)	0.07 (0.02,0.14)	0.10 (0.04,0.18)	1.567	0.117
Cr (μmol/L)	77.43 ± 15.59	76.95 ± 17.37	0.245	0.807
FBG (mmol/L)	7.64 ± 1.92	7.83 ± 2.28	0.778	0.437
ALT (U/L)	31 (20,44)	35 (23,48)	0.998	0.321
AST (U/L)	27 (18,40)	31 (21,44)	1.065	0.287
TBIL (μmol/L)	12.2 (8.3,16.5)	13.2 (9.1,17.5)	0.821	0.412
BUN (mmol/L)	5.7 (4.1,7.4)	6.2 (4.5,8.1)	1.287	0.198
APACHE II score (points)	19.53 ± 5.28	24.14 ± 5.04	7.342	<0.001

Variables such as mechanical ventilation, endovascular therapy, and complications (stroke-associated pneumonia, symptomatic cerebral edema, gastrointestinal bleeding) were documented for descriptive comparison between groups. These post-admission variables were not included as candidate predictors in the nomogram to avoid outcome leakage, as they occur after the baseline prediction time point (ICU admission).

### 3.2 Multivariate logistic regression analysis of the influencing factors related to death in patients with severe cerebral infarction

All parameters that registered a *p-*value below 0.1 in univariate testing were entered into the multivariable logistic regression model for further analysis. The dependent variable was assigned as follows: survival group = 0 and death group = 1. The independent variables, including age, NIHSS score, NLR, hs-CRP, IL-6 levels, and APACHE II scores, were included as continuous variables using their actual values. In addition, endovascular therapy was forced into the model as a covariate to adjust for potential confounding factors, regardless of its univariate significance. VIF diagnostics were used to examine collinearity. All independent variables had VIF scores lower than 5, proving that no obvious multicollinearity existed in the dataset. In the forward stepwise selection process, APACHE II was retained in the final model, whereas age and GCS were not, indicating that APACHE II provided a stronger composite prognostic signal in this specific cohort. All variance inflation factors (VIFs) in the final model were < 2.0, further supporting the absence of problematic multicollinearity. Univariate logistic regression identified age, NIHSS score, NLR, hs-CRP, IL-6 levels, and APACHE II score as as factors associated with mortality among patients with severe cerebral infarction. Further multivariable logistic regression analyses confirmed that the five indicators above were independent risk factors for fatal outcomes in this patient population (all *p* < 0.05). Details are shown in Tables 2, 3.

**Table 2:** Univariate logistic regression analysis of factors associated with death in patients with severe cerebral infarction.

Factor	*β*	*SE*	*Wald*	*p*	*OR*	95% *CI*
Age	0.023	0.011	4.247	0.039	1.023	(1.001–1.046)
NIHSS score	0.137	0.033	17.367	<0.001	1.147	(1.075–1.224)
NLR	0.392	0.060	42.219	<0.001	1.480	(1.315–1.666)
hs-CRP	0.086	0.017	25.994	<0.001	1.090	(1.055–1.127)
IL-6	0.049	0.010	24.941	<0.001	1.050	(1.030–1.071)
APACHE II	0.176	0.028	40.218	<0.001	1.193	(1.130–1.260)

**Table 3:** Multivariate logistic regression analysis of the influencing factors related to death in patients with severe cerebral infarction.

Factor	*β*	*SE*	*Wald*	*p*	*OR*	95% *CI*
Age	0.023	0.015	2.351	0.125	1.023	(0.994–1.053)
NIHSS score	0.098	0.041	5.712	0.017	1.103	(1.018–1.195)
NLR	0.475	0.079	36.148	<0.001	1.608	(1.378–1.877)
hs-CRP	0.098	0.023	18.154	<0.001	1.103	(1.054–1.155)
IL-6	0.050	0.013	14.793	<0.001	1.051	(1.025–1.078)
Endovascular therapy	−0.062	0.311	0.040	0.841	0.940	(0.511 ~ 1.730)
APACHE II	0.176	0.035	24.875	<0.001	1.193	(1.113–1.278)

### 3.3 The combined predictive value of related influencing factors for the mortality of patients with severe cerebral infarction

The combined use of the NIHSS score, hs-CRP, IL-6, NLR, and APACHE II scores in predicting 30-day mortality in patients with severe cerebral infarction had a higher AUC value than individual tests (Z = 6.879, 6.454, 6.693, 5.480, 5.407, *p* < 0.05). Details are shown in Table 4 and Figure 2.

**Table 4:** Predictive value of combined related influencing factors for death in patients with severe cerebral infarction.

Item	AUC	Standard error	95% *CI*	*p*	Youden index	Cut-off value	Sensitivity (%)	Specificity (%)
NIHSS	0.656	0.032	0.600–0.708	<0.001	0.277	>21 points	70.59	57.14
hs-CRP	0.686	0.032	0.631–0.737	<0.001	0.304	>33.6 mg/L	47.06	83.33
IL-6	0.670	0.033	0.614–0.722	<0.001	0.248	>47.6 pg./mL	67.65	57.14
NLR	0.741	0.031	0.688–0.788	<0.001	0.385	>9.2	65.69	72.86
APACHE II	0.736	0.030	0.683–0.784	<0.001	0.390	>22 points	65.69	73.33
Combined	0.888*	0.020	0.848–0.921	<0.001	0.620	>0.236	86.27	75.71

*indicates *p* < 0.05 compared with individual detection.

**Figure 2:**
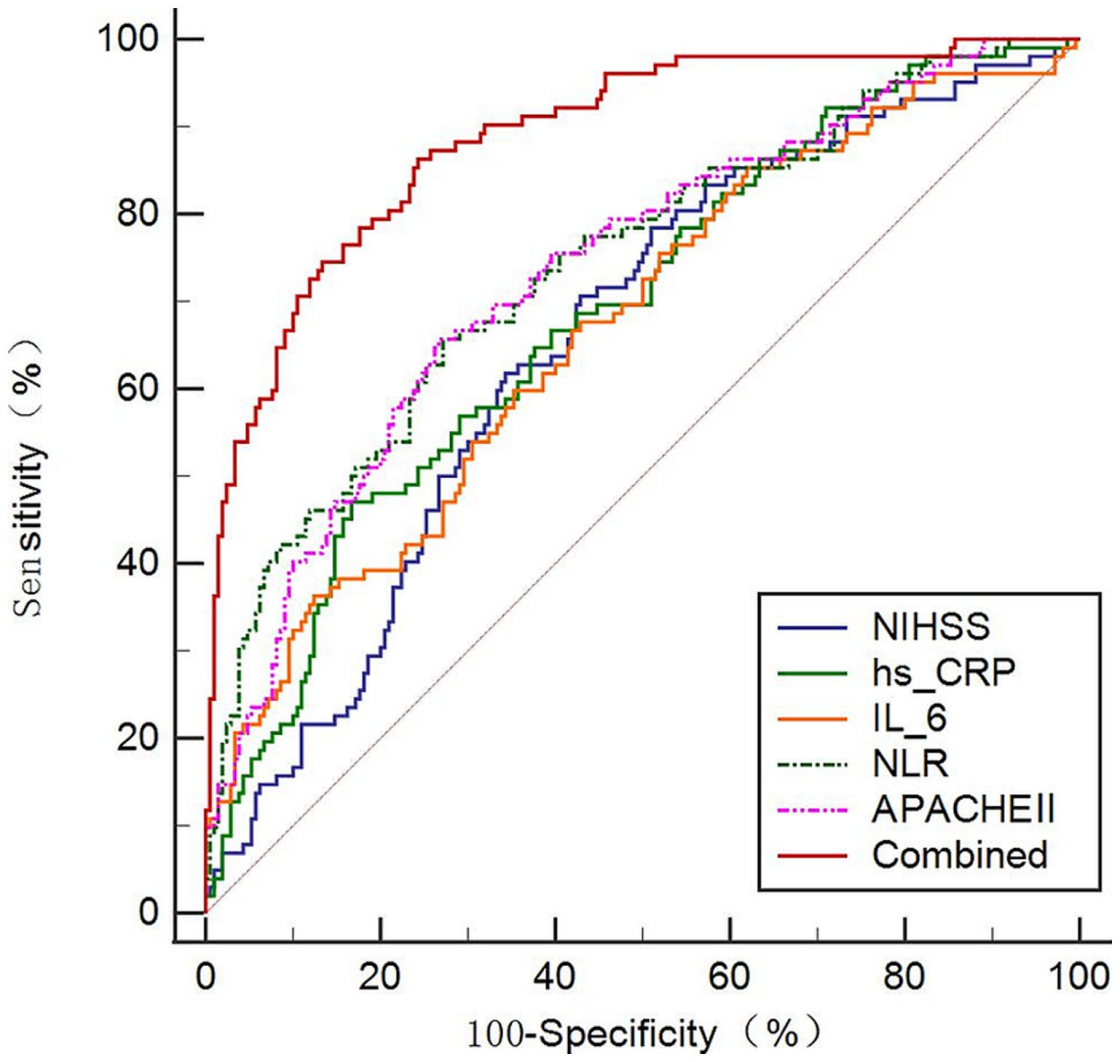
ROC curve illustrating the combined predictive value of relevant influencing factors for the mortality of patients with severe cerebral infarction.

### 3.4 Comparison of clinical data between the model group and the validation group

The baseline characteristics of the derivation and validation cohorts are compared in Table 5. Standardized mean differences (SMDs) were calculated to assess the balance between the two cohorts. All key variables had SMDs < 0.1, indicating a good balance between the cohorts and supporting the transportability of the model across populations (Table 5).

**Table 5:** Comparison of clinical data between the model and the validation groups.

Item	Model group (*n* = 312)	Validation group (*n* = 134)	SMD
Age (years)	66.51 ± 11.03	66.93 ± 10.90	0.038
Male	182 (58.33)	76 (56.72)	0.033
BMI (kg/m^2^)	24.32 ± 3.06	24.84 ± 3.31	0.163
History of hypertension	220 (70.51)	90 (67.16)	0.072
History of diabetes mellitus	115 (36.86)	46 (34.33)	0.053
History of coronary heart disease	79 (25.32)	37 (27.61)	0.052
History of atrial fibrillation	68 (21.79)	36 (26.87)	0.119
Smoking history	136 (43.59)	55 (41.04)	0.052
Drinking history	105 (33.65)	39 (29.10)	0.099
NIHSS score (points)	21.01 ± 4.05	21.19 ± 4.17	0.044
GCS score (points)	8.56 ± 2.78	8.60 ± 2.64	0.015
Mechanical ventilation	155 (49.68)	72 (53.73)	0.081
Endovascular therapy	89 (28.53)	35 (26.12)	0.055
Infarct location			0.102
Anterior circulation	213 (68.27)	85 (63.43)	
Posterior circulation	99 (31.73)	49 (36.57)	
Complications			
Stroke-associated pneumonia	129 (41.35)	53 (39.55)	0.037
Symptomatic cerebral edema	115 (36.86)	43 (32.09)	0.101
Gastrointestinal bleeding	51 (16.35)	25 (18.66)	0.061
WBC (×10^9^/L)	11.27 ± 2.77	11.12 ± 2.72	0.055
MON (×10^9^/L)	0.69 ± 0.19	0.70 ± 0.19	0.053
PLT (×10^9^/L)	218.12 ± 53.66	217.37 ± 52.86	0.014
NLR	8.44 ± 2.64	8.25 ± 2.61	0.073
hs-CRP (mg/L)	28.61 ± 8.32	29.78 ± 8.36	0.141
IL-6 (pg/mL)	48.19 ± 13.73	48.51 ± 13.12	0.024
PCT (ng/mL)	0.08 (0.03, 0.15)	0.09 (0.04, 0.16)	0.077
Cr (μmol/L)	77.71 ± 16.92	78.72 ± 18.57	0.057
FBG (mmol/L)	7.71 ± 1.96	7.72 ± 1.78	0.005
ALT (U/L)	32 (21, 45)	33 (22, 46)	0.069
AST (U/L)	28 (19, 41)	29 (20, 42)	0.058
TBIL (μmol/L)	12.5 (8.6, 16.8)	12.8 (8.8, 17.0)	0.063
BUN (mmol/L)	5.8 (4.2, 7.5)	5.9 (4.3, 7.6)	0.068
APACHE II score (points)	21.14 ± 5.77	21.39 ± 6.10	0.042

SMD, Standardised Mean Difference. SMD < 0.1 indicates good balance between cohorts; SMD between 0.1 and 0.3 indicates acceptable balance.

### 3.5 Based on the logistic regression analysis, a risk prediction nomogram for severe cerebral infarction was developed

Based on the logistic regression analysis, a prediction nomogram for the risk of 30-day mortality in patients with severe cerebral infarction was established. The nomogram included five variables: NIHSS score, NLR, hs-CRP, IL-6, and APACHE II score. Among them, NLR had a relatively high weight in the nomogram, indicating that it plays an important role in the risk prediction (Figure 3).

**Figure 3:**
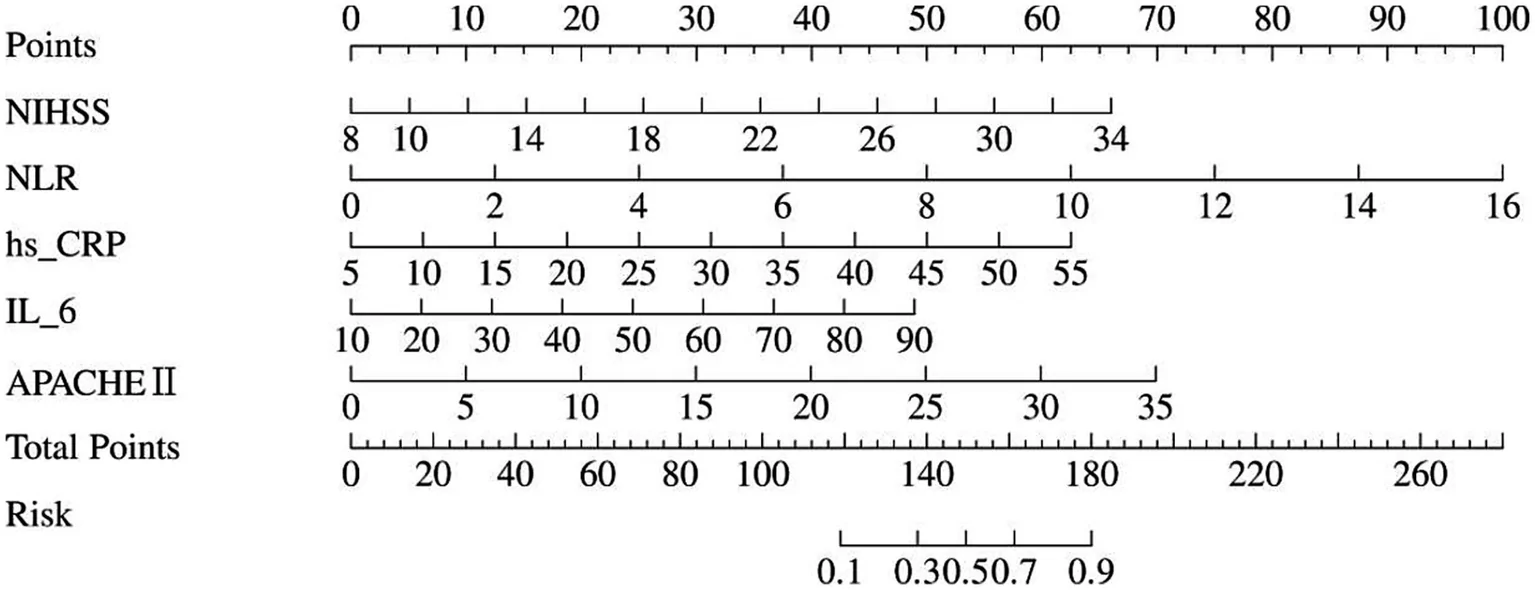
Risk prediction nomogram for severe cerebral infarction based on the logistic regression analysis.

The ROC curve for the derivation cohort showed an apparent AUC of 0.888 (Figure 4A). After optimism correction using 1,000 bootstrap resamples, with the entire stepwise selection process repeated in each resample, the optimism-corrected AUC was 0.856 (Figure 4B), indicating that the model maintained a good discriminative ability with only modest optimism. The calibration slope was 0.94, the calibration intercept was −0.03, and the Brier score was 0.131, indicating good overall calibration and predictive accuracy of the model. The calibration plot (Figure 5A) shows a satisfactory agreement between the predicted and observed outcomes. The Hosmer–Leme show goodness-of-fit test yielded a *p*-value of 0.211, which, together with the calibration slope and intercept, supported adequate model calibration. The calibration curve for the validation cohort is shown in Figure 5B. This part also reflected favorable calibration, where the predicted outcomes were in good agreement with the actual records. The fit test yielded *p* = 0.877, indicating that the model fitted well in this group. DCA showed that applying this nomogram within a risk threshold of 0.1 to 0.8 can bring a clinical net benefit (Figures 6A,B).

**Figure 4:**
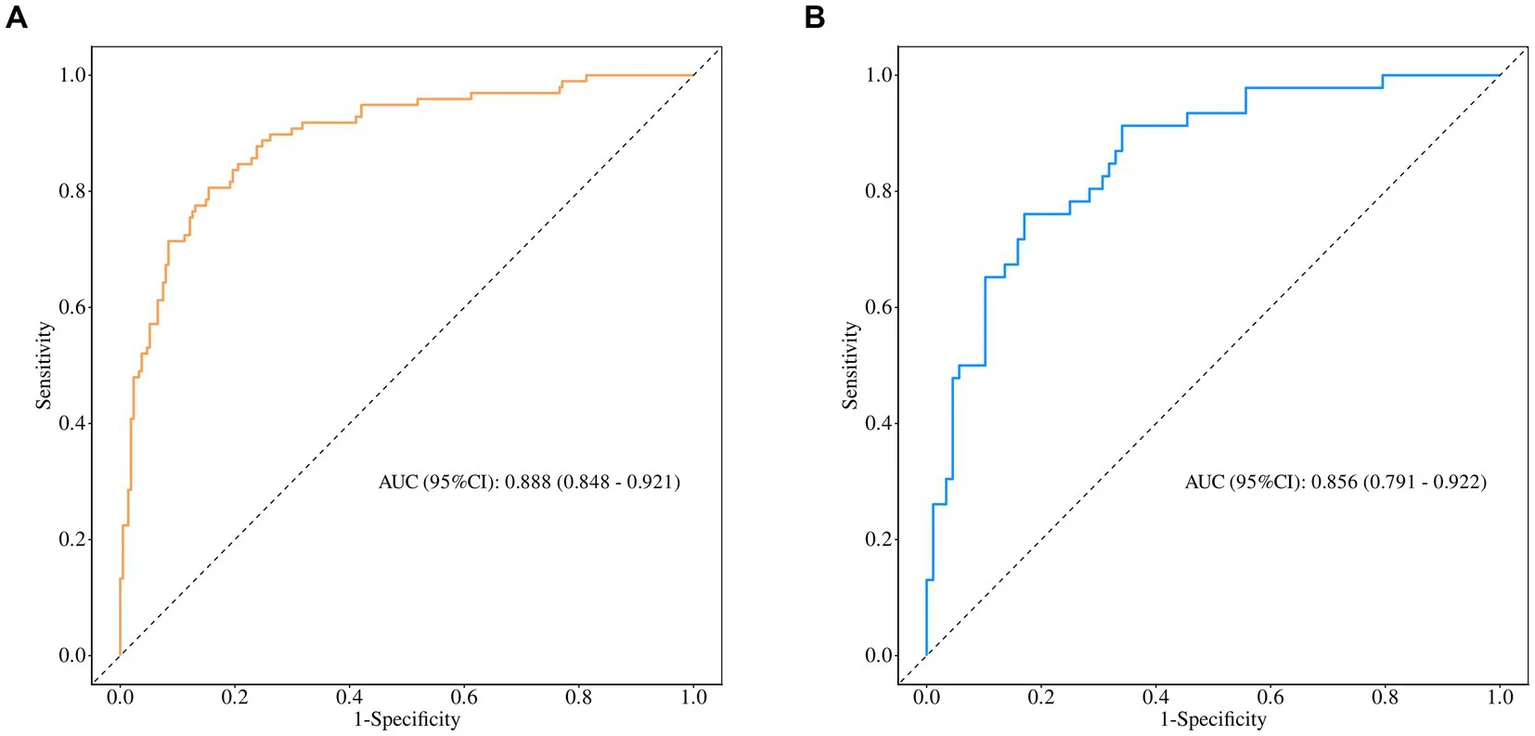
ROC curve. **(A)** Model group. **(B)** Validation group.

**Figure 5:**
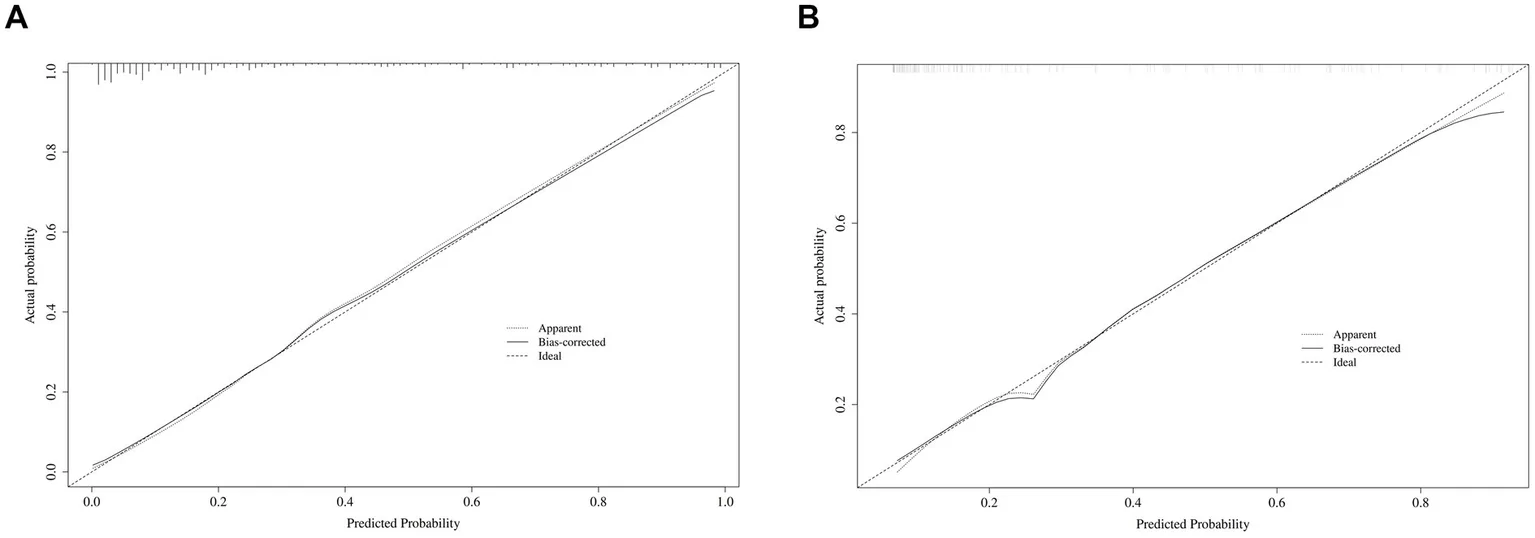
Calibration curve. **(A)** Model group. **(B)** Validation group.

**Figure 6:**
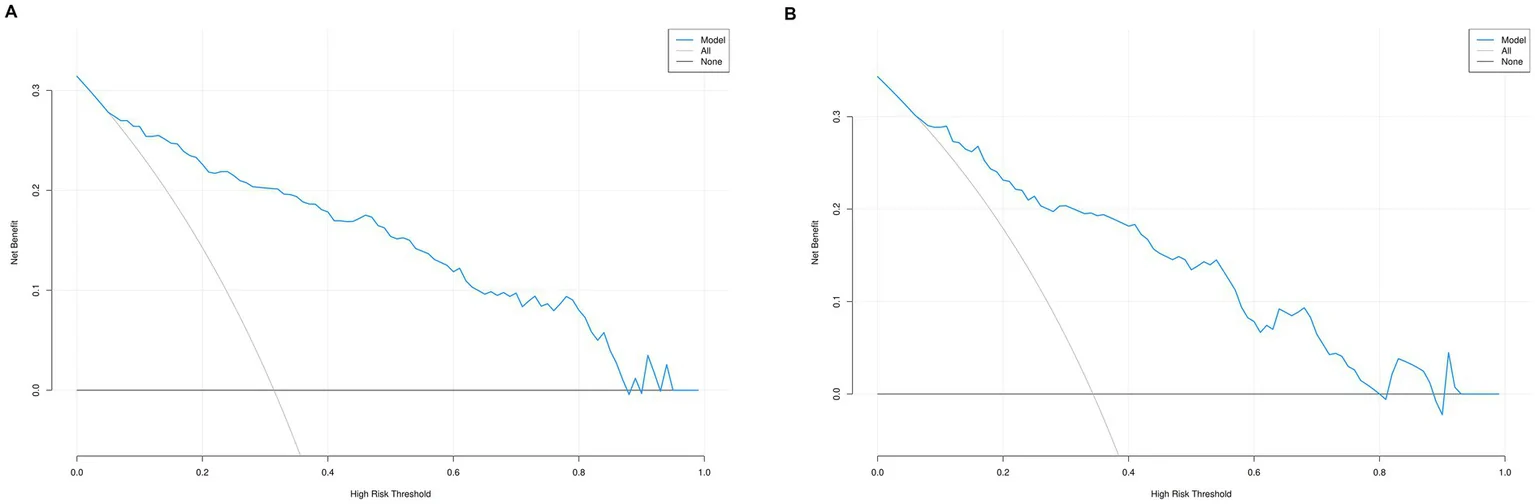
Decision curve. **(A)** Model group. **(B)** Validation group.

### 3.6 Sensitivity analysis: pre-specified full model

In the clinically pre-specified full model, all variables were retained. The analysis confirmed that NIHSS (*OR* = 1.101, *p* = 0.019), NLR (*OR* = 1.599, *p* < 0.001), hs-CRP (*OR* = 1.101, *p* < 0.001), IL-6 (*OR* = 1.049, *p* < 0.001), and APACHE II (*OR* = 1.189, *p* < 0.001) remained statistically significant independent risk factors. Age (*OR* = 1.020, *p* = 0.142) and GCS (*OR* = 0.964, *p* = 0.251) were not significant. The full model demonstrated an apparent AUC of 0.889 (95%CI: 0.849–0.922), which was nearly identical to the stepwise model’s AUC of 0.888. After bootstrap optimism correction, the AUC was 0.857, further supporting the model’s stability.

## 4 Discussion

A retrospective review was conducted on clinical information of 312 patients with severe cerebral infarction. This study investigated the predictive potential of combining inflammation-related markers and classic scoring systems for 30-day fatal risk. The analysis results demonstrated that NIHSS, NLR, hs-CRP, IL-6, and APACHE II scores were key independent risk determinants for 30-day mortality. The combined prediction model built on these indices presented a satisfactory predictive performance, which can support early clinical risk stratification with credible theoretical evidence.

The APACHE II score is the most widely used prognostic assessment tool for critically ill patients in clinical practice. It considers the patient’s acute physiological indicators, chronic health status, and age factors and can comprehensively reflect the severity of the patient’s overall condition (10). The results of this study show that the APACHE II score is one of the independent risk factors for 30-day mortality in patients with severe cerebral infarction (*OR* = 1.193, 95% *CI*: 1.113–1.278). The individual prediction of death had an AUC of 0.736, which is consistent with the results of related studies at home and abroad (11). However, the single APACHE II score mainly reflects the patient’s overall physiological condition and is difficult to specifically reflect the local pathological and physiological processes of cerebral infarction, especially the key role of neuroinflammatory responses in disease progression. Therefore, its predictive efficacy requires improvement.

A notable finding is that APACHE II was retained as a significant predictor in the final model, while age and GCS were not. We attribute this to the conceptual redundancy and the composite nature of the APACHE II score. Although age and GCS are components of APACHE II, the score itself is a far more comprehensive measure of acute physiological derangement. It integrates objective parameters of vital organ function (e.g., cardiovascular, respiratory, renal) and chronic health conditions, which are key determinants of survival in critically ill patients but are not captured by age or GCS alone. Our analysis shows that the APACHE II score had a stronger and more direct association with 30-day mortality (*OR* = 1.193, AUC = 0.736) than either age (*OR* = 1.023, AUC = 0.560) or GCS (*OR* = 0.950, AUC = 0.531).

Over the past several years, a wealth of clinical research has proven that inflammatory processes exert a profound influence on the onset, progression, and prognostic outcomes of cerebral infarction (12). Once cerebral infarction sets in, neuroinflammation triggered by local ischemia and hypoxia leads to the breakdown of the blood–brain barrier, increased brain edema, neuronal apoptosis, and secondary brain injury, which in turn affects the clinical outcome of patients (13). NLR is a new type of composite inflammatory indicator that integrates the innate immune information mediated by neutrophils and the adaptive immune information mediated by lymphocytes and can comprehensively reflect the immune inflammatory status of the body (14). The results of this study show that NLR is an independent risk factor for 30-day mortality in patients with severe cerebral infarction (OR = 1.611, 95% CI: 1.380–1.880), and its individual prediction of death has an AUC of 0.741, which is higher than other single inflammatory indicators, and has the highest weight in the nomogram model. As an integrative inflammatory indicator, an elevated NEU in NLR reflects intense innate immune activation after acute ischemia and aggravates the disruption of the blood–brain barrier and cerebral edema. A decreased LYM indicates an immune-suppressive state induced by stroke, which increases the risk of secondary infection. At the same time, it captures both the pro-inflammatory and immunosuppressive dimensions, making the NLR have a stronger independent effect in predicting 30-day mortality in patients with severe cerebral infarction. Nevertheless, it is important to acknowledge that these inflammatory biomarkers are non-specific and may be influenced by concurrent subclinical inflammatory conditions. To address this, we deliberately excluded patients with recent infections, autoimmune disorders, or malignancies, thereby minimizing potential confounding from pre-existing systemic inflammation. However, this exclusion also limits the generalizability of our model to unselected populations, and clinicians should consider the potential for false-positive elevations of these markers in patients with concurrent inflammatory conditions when applying the nomogram in practice. Che et al. (15) found that an elevated NLR is closely related to the poor prognosis of patients with severe cerebral infarction. Patients with NLR > 8.64 had a significantly increased 30-day mortality risk, which is consistent with the results of this study. The optimal cutoff value of NLR determined in this study was 9.216, which is close to the result of this study, further confirming the important value of NLR in the prognosis assessment of severe cerebral infarction.

As a classic acute-phase protein, hs-CRP is secreted by the liver and serves as a highly sensitive biomarker for assessing inflammatory reactions in the body (5). The results of this study show that hs-CRP is an independent risk factor for 30-day mortality in patients with severe cerebral infarction (*OR* = 1.104, 95% CI: 1.055–1.156), which is consistent with the results of Ni et al. (16). A total of 183 patients diagnosed with acute ischemic stroke were recruited for this study. After adjusting for a range of confounding variables, multivariable logistic regression models confirmed that the hsCRP/HDL-C ratio could independently predict adverse clinical outcomes at the 3-month follow-up. This study also confirmed that hs-CRP levels were significantly correlated with stroke and poor prognosis.

IL-6 is a multifunctional cytokine and an upstream factor that drives CRP production. Its level rises earlier than that of CRP and can more accurately reflect the degree of inflammatory response in the body (17). The results of this study show that IL-6 is also an independent risk factor for 30-day mortality in patients with severe cerebral infarction (*OR* = 1.052, 95% CI: 1.026–1.079), and its optimal cut-off value is 47.6 pg./mL. Tang et al. (18) reported that increased IL-6 levels are an independent prognostic factor for a negative prognosis in patients diagnosed with acute ischemic stroke. This result is consistent with the results of the present study.

Regarded as the well-accepted gold standard, the NIHSS is widely applied to assess neurological deficit severity among patients with acute ischemic stroke. This scale can effectively mirror the severity of the cerebral lesion and its lesion scope (19). Our statistical analyses further proved that NIHSS score acts as a standalone risk determinant for 30-day mortality in patients with severe cerebral infarction (*OR* = 1.107, 95% *CI*: 1.020–1.201). Notably, in this study, the univariate analysis showed that age was related to 30-day mortality in patients with severe cerebral infarction (*p* = 0.038), but in the multivariate analysis, age was no longer an independent risk factor (*p* = 0.109). Analyzing the reasons, it may be because the influence of age on prognosis is mediated by other more direct factors, such as the degree of neurological impairment (NIHSS score) and the degree of systemic inflammatory response (NLR, hs-CRP, IL-6). This result suggests that for elderly patients with severe cerebral infarction, negative treatment decisions should not be made solely based on age; rather, the patient’s neurological condition and inflammatory response level should be comprehensively evaluated (20).

Based on previous studies, this study further integrated three representative inflammatory indicators (NLR, hs-CRP, and IL-6) with the NIHSS and APACHE II scores to construct a 30-day mortality risk prediction nomogram model specifically for patients with severe cerebral infarction. Compared with previous single indicators or simple scoring systems, our model simultaneously captures three complementary pathophysiological dimensions of severe cerebral infarction, resulting in a significant improvement in the predictive efficacy. First, the NIHSS score provides a direct and specific measure of the clinical severity and extent of focal neurological injury. Second, the inflammatory markers (NLR, hs-CRP, and IL-6) reflect the systemic inflammatory cascade and immune dysregulation that follows cerebral ischemia, which is not captured by NIHSS. Specifically, NLR integrates both the pro-inflammatory (neutrophil) and immunosuppressive (lymphocyte) axes; hs-CRP signals the hepatic acute-phase response to inflammation; and IL-6 is an upstream driver of this response, offering a more dynamic and earlier indicator of the inflammatory state. These markers provide complementary information on neuroinflammatory processes. Third, the APACHE II score offers a broad assessment of the patient’s overall physiological reserve and severity of multiorgan dysfunction, independent of neurological and inflammatory status. By combining these three dimensions, our model provides a holistic view of the patient’s condition, accounting for local neurological injury, systemic inflammatory response, and overall physiological tolerance, which is essential for accurate risk stratification in these complex patients.

The AUC of this combined model was 0.888 for predicting 30-day all-cause mortality among patients with severe cerebral infarction. This model outperformed all standalone predictive parameters, with significant differences. This indicates that integrating inflammatory indicators can significantly improve the model’s predictive accuracy and more comprehensively reflect the complex pathophysiological process of severe cerebral infarction (21). Furthermore, we transformed the combined model into an intuitive nomogram. Clinicians can quickly obtain the individualized mortality risk prediction value of patients through simple calculations, thereby better guiding clinical treatment decisions. For high-risk patients, enhanced monitoring can be implemented, and more aggressive anti-inflammatory treatment, dehydration, and reduction of intracranial pressure can be adopted at an early stage to reduce the mortality rate. For low-risk patients, excessive medical treatment can be avoided, and medical resources can be conserved (22).

This study has several limitations. First, this was a single-center retrospective study, which may have introduced selection bias. Second, although we adjusted for the presence of endovascular therapy in our multivariate model, we did not have access to granular data on the time-to-treatment, specific technique used, success of recanalization, or post-procedural complications, all of which could influence the prognosis. Third, we acknowledge the well-recognized limitations of using stepwise logistic regression for variable selection, including potential model instability, overfitting, and biased coefficient estimates. We recommend that future, larger prospective studies confirm our findings using more robust techniques, such as penalized regression methods (e.g., LASSO), which offer superior variable selection and shrinkage properties. Fourth, this study only detected the inflammatory indicators within 24 h after the patient’s admission and did not dynamically observe the changing trend of the inflammatory indicators. Dynamic monitoring of inflammatory indicators may be more helpful in assessing changes in the patient’s condition and treatment response. Finally, this study did not explore the impact of anti-inflammatory treatment on the prognosis of patients with severe cerebral infarction. Future investigations should consist of multicenter prospective cohort studies with larger participant pools. We will conduct serial monitoring of inflammatory biomarkers at various time points post-admission. Additional analyses will assess the efficacy of early anti-inflammatory therapy on clinical outcomes in subgroups stratified by the severity of inflammatation. These follow-up attempts will serve to validate our current findings and generate evidence to facilitate more targeted and individualized patient management in routine care.

## 5 Conclusion

NIHSS score, NLR, hs-CRP, IL-6, and APACHE II scores were independent risk factors for 30-day mortality in patients with severe cerebral infarction. The combined prediction model constructed based on these indicators has high predictive efficacy and can provide a scientific basis for the early identification of high-risk patients and the formulation of individualized treatment plans for clinical practice. However, the methodological limitations of stepwise regression should be acknowledged, and future studies using penalized regression techniques are warranted to further validate these findings.

## Data Availability

The original contributions presented in the study are included in the article/supplementary material, further inquiries can be directed to the corresponding author.
